# Development of the Measurement of Lateral Electron Density (MOLE) Probe Applicable to Low-Pressure Plasma Diagnostics

**DOI:** 10.3390/s22155487

**Published:** 2022-07-22

**Authors:** Si-jun Kim, Sang-ho Lee, Ye-bin You, Young-seok Lee, In-ho Seong, Chul-hee Cho, Jang-jae Lee, Shin-jae You

**Affiliations:** 1 Applied Physics Lab for PLasma Engineering (APPLE), Department of Physics, Chungnam National University, Daejeon 34134, South Korea; sjk@o.cnu.ac.kr (S.-j.K.); esangho35@kimm.re.kr (S.-h.L.); 201500963@o.cnu.ac.kr (Y.-b.Y.); lerounsukre@o.cnu.ac.kr (Y.-s.L.); showing123@o.cnu.ac.kr (I.-h.S.); paulati@o.cnu.ac.kr (C.-h.C.); 2 Department of Plasma Engineering, Korea Institute of Machinery and Materials (KIMM), Daejeon 34104, South Korea; 3 Samsung Electronics, Hwaseong-si 18448, South Korea; jangjae2.lee@samsung.com; 4 Institute of Quantum Systems (IQS), Chungnam National University, Daejeon 34134, South Korea

**Keywords:** plasma diagnostics, non-invasive electron density measurement, planar microwave probes, plasma monitoring

## Abstract

As the importance of measuring electron density has become more significant in the material fabrication industry, various related plasma monitoring tools have been introduced. In this paper, the development of a microwave probe, called the measurement of lateral electron density (MOLE) probe, is reported. The basic properties of the MOLE probe are analyzed via three-dimensional electromagnetic wave simulation, with simulation results showing that the probe estimates electron density by measuring the surface wave resonance frequency from the reflection microwave frequency spectrum (S${}_{11}$). Furthermore, an experimental demonstration on a chamber wall measuring lateral electron density is conducted by comparing the developed probe with the cutoff probe, a precise electron density measurement tool. Based on both simulation and experiment results, the MOLE probe is shown to be a useful instrument to monitor lateral electron density.

## 1. Introduction

Plasma applications in modern technologies cover numerous fields such as material fabrication, nuclear fusion, medical treatment, agriculture, and catalysis [1,2,3,4]. Among them, plasma has played a key role in the state-of-the-art processing of semiconductor fabrication, particularly in ultra-high-aspect ratio etching, atomic layer etching, and deposition, since plasma has chemically and physically active species [2,5].

Recently, to improve throughput and productivity, process monitoring technology has become significant because the price of a patterned wafer has steadily grown and loss by unstable process is no longer negligible [6,7,8,9]. Particularly, advanced process control (APC), which is a process-tuning technology based on real-time signals from monitoring devices, has attracted strong interest from industry [10,11]. The APC gathers monitoring data in real-time and diagnoses whether plasma processing steps are normal or not based on post-process algorithms [12,13,14]. Non-invasive measurement devices create tremendous monitoring data such as optical emission spectra, voltages applied and current flowing through the discharge electrode or antenna, capacitor positions in an impedance matcher, throttle valve positions, gas flow rates, and plasma parameters such as electron density and temperature [13,15,16,17,18].

Electron density is a significant factor among these monitoring parameters since it is believed to be directly related to processing time and quality [2,5,10,11,17,18]. To measure electron density non-invasively, various diagnostic methods have been developed, including actinometry and the line ratio method by analyzing optical emission spectra [6,19,20], laser Thomson scattering by measuring scattered laser light [20,21], and planar microwave probes by analyzing reflected or transmitted microwave signals [18,22,23]. They all have been frequently employed in the research field but some optical and laser methods have limitations for industrial application, as follows. First, the actinometry and line ratio approaches are only applicable in a narrow processing window, while the laser Thomson scattering method requires a large and stable space to generate the laser and fine-tune its sensitivity. Second, both optical and laser methods are strongly affected by any contamination of the viewport [24]. On the other hand, planar microwave probes are not affected by contamination of the microwave antenna [25] and only slightly perturb the processing plasma [26]. Hence, the planar microwave probe is seen as a promising electron density monitoring device, as evidenced by numerous probe designs as follows.

Based on the plasma cutoff phenomenon, which was firstly employed in a microwave interferometry measuring phase shift due to the plasma cutoff for measuring the line-integrated electron density [27,28], You et al. developed the planar cutoff probe (PCP), which measures the cutoff frequency in the transmission microwave frequency spectrum (S${}_{21}$), which is defined as transmitted power of port 2 over radiated power from port 1 in a frequency spectrum [10,11,18,25]. They proposed the planar cutoff probe [11], developed it as a real-time monitoring instrument [17], and further optimized and analyzed the PCP [10,18]. Sugai et al. developed the planar curling probe (CP), based on the quarter-wave resonance (QWR) on a curling antenna, that measures the shift of QWR frequency induced by plasma in the reflection microwave frequency spectrum (S${}_{11}$), which is defined as reflected power of port 1 over radiated power from port 1 in a frequency spectrum [22,29,30,31]. They deeply analyzed the CP [29,30,31], developed it as a real-time monitoring instrument for electron density as well as film thickness [22]. With similar principle of the CP, Beckers et al. have developed microwave cavity resonance spectroscopy (MCRS), that measures cavity resonance frequency shift by plasma in S11 spectrum [32,33,34]. In the recent attempt, they demonstrated operation of the MCRS with the wall-mounted invasive antenna. Brinkmann et al. developed the planar multipole resonance probe (pMRP) based on dipole resonance; this probe measures multipole resonance frequencies in S${}_{11}$ [23,35,36,37,38]. They introduced the pMRP [36], developed it as a monitoring sensor [37], and further investigated the pMRP [23,38]. Among those probes, the MCRS and microwave interferometry show wide measurement range and short time resolution, whereas there is lack of study for measurement limitations of the PCP, the CP, and the pMRP; those planar probes would be deeply investigated in terms of its detection limitations. Although these planar probes all show high measurement accuracy, the probe module size and design are bulky and complex, respectively.

A small and simple probe size is desirable for the following reasons. First, a small probe design lowers plasma perturbations as well as thermal damage on the probe antenna from the plasma. Recently, high ion fluxes and energy have been generated with high average power (10–30 kW), so thermal damage issues should be considered [39,40]. Second, a simple probe design expands its applicability, such as enabling installation into an electrostatic chuck (ESC) as well as on vacuum chamber walls, with improved assembly tolerance. It should be noted that the capability to be installed onto chamber walls in place of an ESC or powered radio-frequency (rf) electrode has various advantages: addressing rf noise issues induced by high power application, minimizing process perturbation (with no requirement for any modification of an ESC or rf electrode before probe insertion) [17,18], and lowering the thermal damage from high-energy ions and neutrals created near the rf electrode.

To realize such advantages, this paper proposes the measurement of lateral electron density (MOLE) probe. As the probe can be made by merely cutting an RG-401 coaxial cable, it represents the smallest and simplest design among planar microwave probes. For the principle, the MOLE probe is based on the surface wave resonance (SWR) at the plasma–sheath interface and measures the SWR frequency in S${}_{11}$. Here, a sheath is an ion space charge region either covering materials immersed into plasma or plasma itself and is caused by mobility difference of electron and ions. In fact, the MOLE probe shares the same operation physics as the plasma absorption probe (PAP) [41] and the plasma transmission probe (PTP) [42]. The difference compared with the PAP will be discussed in the next section. Here, we briefly explain the difference between the PTP and the MOLE probe: the former measures the SWR frequency in S${}_{21}$, but the latter does so in S${}_{11}$ [42]. This fact brings about two effects: first, the PTP requires a receiving antenna but the MOLE probe does not. Second, the sensitivity of the MOLE probe is higher than that of the PTP; the background signal level is high enough to be comparable to the SWR signal since the radiating and receiving antennae of the PTP are adjacent through a dielectric medium. Furthermore, the SWR frequency is closer to the ideal SWR frequency for the MOLE probe than for the PTP, and thus the MOLE probe can more precisely measure the SWR frequency.

The remainder of this paper is as follows. In the second section, the simulation method to verify the operation physics of the proposed probe is discussed. In the third section, an experimental demonstration of the MOLE probe is described and analyzed. Finally, in the conclusion section, a summary of this paper is provided.

## 2. Three-Dimensional Electromagnetic Wave Simulation Analysis

### 2.1. Simulation Details

To analyze the basic properties of the MOLE probe, the high-frequency time-domain solver provided by CST Microwave Suite was utilized. It solves Maxwell’s equations in three-dimensional (3D) space via the finite-difference time-domain method for the typical microwave range, from several MHz to several tens of GHz. This simulation approach has been widely used to analyze and optimize microwave probes such as the planar cutoff probe, planar curling probe, and planar multipole resonance probe; related simulation details can be found in [10,23,31,35].

**Figure 1:**
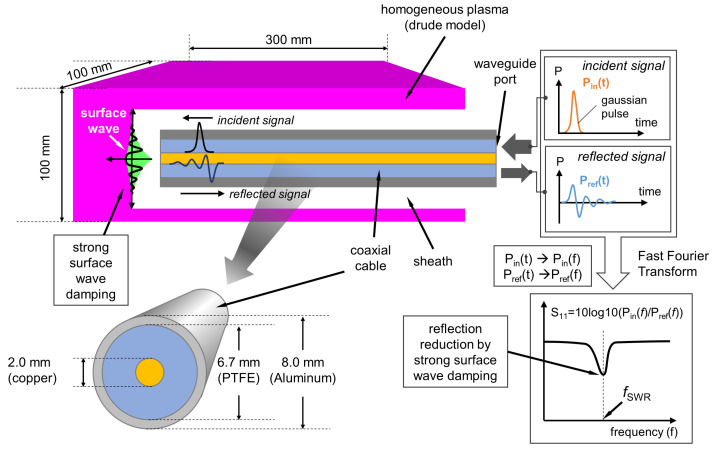
Schematic diagram for the simulation configuration.

Figure 1 shows a schematic diagram for the simulation configuration. A coaxial cable is partially immersed into a rectangular plasma of dimensions 100 × 100 × 300 mm${}^{3}$. The plasma was assumed as a dispersive dielectric material, or more specifically the Drude model provided by the solver. One can find details on the Drude model in [10]. Briefly, based on the Drude model, the plasma dielectric constant can be calculated by the electron density ($n_{e}$), collision frequency ($\nu_{m}$), and microwave frequency (*f*). Here, $\nu_{m}$ is calculated with the same assumptions as in [10], and the sheath is assumed as a vacuum (the relative dielectric constant of the sheath is unity). In fact, the sheath is an ion-space charge region (rigorously not a vacuum) but in terms of microwave, of which frequency is much higher than ion plasma oscillation frequency, ions are immobile and therefore the sheath can be assumed as a vacuum. In this simulation condition, $n_{e}$, $\nu_{m}$, and sheath width are variables.

The benchmark dimension of the coaxial cable is a RG-401 cable used in this experiment; not exactly the same but similar dimensions for simple numeral. The coaxial cable consists of a cylindrical copper core 2.0 mm in diameter, a polytetrafluoroethylene (PTFE) tube 6.7 mm in outer diameter and 2.0 mm in inner diameter, a relative dielectric constant of 2.1, and an aluminum tube 8.0 mm in outer diameter and 6.7 mm in inner diameter.

A nanosecond Gaussian pulse ($P_{\mathrm{in}}\left(t\right)$) including microwaves from 0 to 10 GHz is applied at the waveguide port, as shown in Figure 1. Then, reflected signals ($P_{\mathrm{ref}}\left(t\right)$) come from the end of the probe, sheath, and plasma in the time domain. As we interested in analysis on the reflected signals in the frequency domain, by using fast Fourier transform, both $P_{\mathrm{in}}\left(t\right)$ and $P_{\mathrm{ref}}\left(t\right)$ are converted into $P_{\mathrm{in}}\left(f\right)$ and $P_{\mathrm{ref}}\left(f\right)$, which are input and reflected powers, respectively, in the frequency domain. Then, S${}_{11}$ is calculated as $10\log_{10}(P_{\mathrm{in}}$(*f*)/$P_{\mathrm{ref}}$(*f*)).

### 2.2. Validation of Surface Wave Resonance

**Figure 2:**
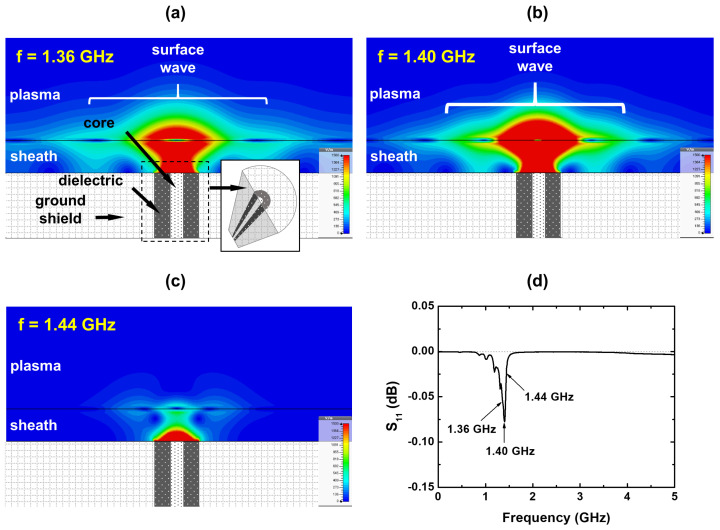
Magnitude of the absolute electric field ($E^{2}=E_{x}^{2}+E_{y}^{2}+E_{z}^{2}$) at different frequencies: (**a**) 1.36 GHz, (**b**) 1.40 GHz, and (**c**) 1.44 GHz, and (**d**) S${}_{11}$ spectrum The electron density is $5\times 10^{10}\;{\mathrm{cm}}^{-3}$, pressure 100 mTorr, and sheath width 5.0 mm. Here, the resonance frequency is 1.40 GHz.

To analyze the basic characteristics of the SWR, a simple simulation model was employed with a thick ground shield 300 mm in diameter and other parameters the same as those in Figure 1. Then, the plasma–sheath interface near the coaxial cable becomes flat and the surface wave propagation on the interface is clearly visualized as shown in Figure 2a–c, which show the magnitude of the absolute electric field with a cross-sectional view at π/2 phase and different frequencies. Based on Figure 2a,b, a surface wave is generated from the plasma–sheath interface. It can be noted that the magnitude of the electric field at a frequency of 1.40 GHz is significantly large, indicating that the surface wave at this frequency is strongly localized at the interface and absorbed by the plasma. This minimizes the amount of the reflected waves. Such a result corresponds to the simulation result that the minimum $S_{11}$ is at 1.40 GHz, as shown in Figure 2d. The surface wave disappears at higher frequencies (>1.40 GHz), as shown in Figure 2c, which implies that the dispersion relation of the surface wave no longer exists above 1.40 GHz and, hence, this frequency indicates the SWR frequency [43]. Furthermore, this frequency is significantly close to the SWR frequency theoretically defined as $f_{\mathrm{SWR}}$ = $f_{\mathrm{pe}}/\sqrt{2}$ [5,43] at an infinite boundary and without collisions, where $f_{\mathrm{pe}}$ is the plasma oscillation frequency ($f_{\mathrm{pe}}=8980\sqrt{n_{e}}$); this theoretical SWR frequency is about 1.42 GHz. It can therefore be concluded that the resonance frequency at which the S${}_{11}$ value is minimum is the SWR frequency.

Moreover, the fact that the electric field strongly localizes at the plasma–sheath interface means that the surface wave resonance frequency includes information on the plasma, the dimensions of which are nearly the same as the diameter of the probe. This implies that the MOLE probe can measure the lateral electron density when it is installed on a chamber wall.

### 2.3. Simulation Results and Discussion

**Figure 3:**
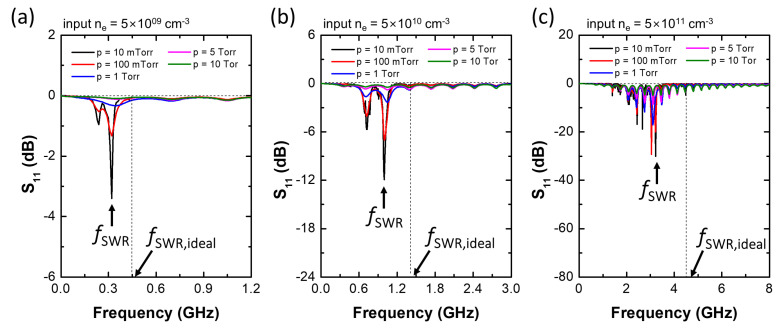
S${}_{11}$ spectra with various pressures (*p*) at a constant sheath width of 0.234 mm and electron densities ($n_{e}$) of (**a**) $5\times 10^{9}\;{\mathrm{cm}}^{-3}$, (**b**) $5\times 10^{10}\;{\mathrm{cm}}^{-3}$, and (**c**) $5\times 10^{11}\;{\mathrm{cm}}^{-3}$.

Through the last section, it can be verified that the SWR induces the minimum S${}_{11}$ value. Now, this section further analyzes the SWR frequency based on the configuration shown in Figure 1. Figure 3 plots the S${}_{11}$ spectra from 0 to 10 GHz with various input electron densities and pressures at a constant sheath width of 0.234 mm. At a low input $n_{e}$ of $5.0\times 10^{9}{\mathrm{cm}}^{-3}$, one can find a sharp resonance peak near 0.3 GHz and multiple peaks above 1 GHz. The former is believed to be the SWR peak and the others due to standing waves formed inside the coaxial cable based on an electric field analysis, which is not included in this paper as it is considered out of scope. As shown in Figure 3a–c, both the SWR frequency and the Q-factor (sharpness of the peak) increase with increasing input $n_{e}$. As mentioned previously, at the SWR condition, the surface wave is localized at the plasma–sheath interface and absorbed by the plasma, leading to an abrupt drop in S${}_{11}$ at the SWR frequency. These wave confinement and absorption effects increase at higher electron densities, with the signal reduction at the SWR frequency becoming stronger.

**Figure 4:**
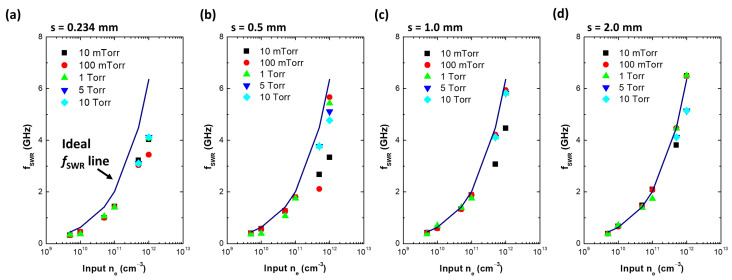
Surface wave resonance frequencies ($f_{\mathrm{SWR}}$) with various pressures and input electron densities at sheath widths of (**a**) 0.234 mm, (**b**) 0.5 mm, (**c**) 1.0 mm, and (**d**) 2.0 mm.

Furthermore, the Q-factor abruptly decreases with increasing pressure, as shown in Figure 3. At high-pressure conditions, electrons barely interact with electromagnetic (E/M) waves due to frequent collisions between argon atoms and electrons, and consequently the wave absorption by the plasma becomes inefficient. As a result, the wave confinement effect decreases and the S${}_{11}$ reduction at the SWR frequency becomes weaker. Figure 4 plots the calculated $f_{\mathrm{SWR}}$ over input $n_{e}$ at various pressures and sheath widths. The dashed line in each plot indicates the ideal SWR frequency as defined by $f_{\mathrm{SWR}}$ = 6349$\sqrt{n_{e}}$. At a thin sheath width (Figure 4a), there is a large difference between the ideal SWR and the calculated $f_{\mathrm{SWR}}$. The maximum discrepancy (defined as $f_{\mathrm{ideal}\mathrm{SWR}}-f_{\mathrm{SWR}}/f_{\mathrm{ideal}\mathrm{SWR}}$) is 36.5% at an $n_{e}$ of $1.0\times 10^{12}\;{\mathrm{cm}}^{-3}$. This is because of the boundary effect, which is not included in the ideal SWR model. At a thin sheath width, the E/M wave damped along the plasma–sheath interface interacts with the plasma as well as with the ground aluminum tube of the coaxial cable. As the sheath width increases, interaction between the E/M wave and the plasma becomes dominant and $f_{\mathrm{SWR}}$ corresponds to the ideal SWR frequency for most pressure conditions, as shown in Figure 4d. Through the E/M wave simulation, it is verified that the sharp resonance peak in the S${}_{11}$ spectrum of the MOLE probe originates from the SWR and values of $n_{e}$ can be inferred by measuring the SWR frequency by the relation $f_{\mathrm{SWR}}=6349\sqrt{n_{e}}$. However, depending on the sheath width, the measurement discrepancy may increase up to 36.5%.

Those simulation results indicate that both detection limit and accuracy largely depend on the sheath width. In the case of floating sheath, as electron density lowered sheath becomes thicker, which leads to a reduction in signal-to-noise ratio since most electromagnetic waves are already reflected on the open termination of the probe edge at thick sheath condition. Meanwhile, at high electron density, the sheath becomes thin, which leads to decreasing accuracy.

### 2.4. Difference between the MOLE Probe and the PAP

**Figure 5:**
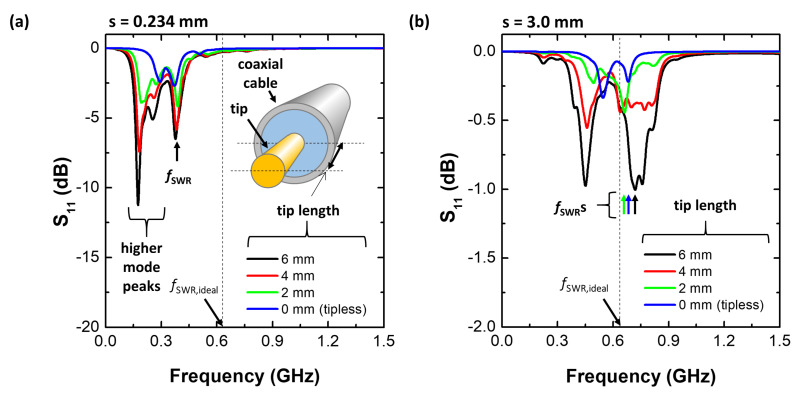
S${}_{11}$ spectra with various tip lengths at a sheath width of (**a**) 0.234 mm and (**b**) 3.0 mm, electron density of $1\times 10^{10}$ cm${}^{-3}$, and pressure of 100 mTorr.

As mentioned in the *Introduction* section, the principle of the MOLE probe is the same as the PAP [41,44,45], except for probe configuration: the PAP has a radiating tip whereas the MOLE probe is tipless, as shown in Figure 1. This difference seems to be trivial, just either *presence* of the tip or *not*, but we found that there is a significant difference in terms of practical use. In general, the S${}_{11}$ spectrum of the PAP shows multiple resonance peaks caused by higher resonance modes as in [41,45] whereas the MOLE probe exhibits just *double* peaks. In fact, these multiple peaks are already addressed in [44] where the sensitive PAP was proposed to minimize higher mode peaks. We found, however, that the sensitive PAP is not effective under different conditions. Figure 5 shows simulated S${}_{11}$ spectra with various tip lengths at different sheath widths of 0.234 mm and 3.0 mm. As shown in Figure 5a, multiple peaks caused by higher mode resonances [45] show higher S${}_{11}$ values compared with the fundamental peak ($f_{\mathrm{SWR}}$). In practical situations where one determines the $f_{\mathrm{SWR}}$ in a measured S${}_{11}$ spectrum, higher mode peaks would result in a large measurement discrepancy of measurement or require additional analytic processes. The higher mode peaks are however suppressed with lessening the tip length and at the tipless condition, S${}_{11}$ spectrum shows clear double peaks or a single peak, depending on conditions. Similarly, at the thick sheath condition (Figure 5b), one can find a complicated S${}_{11}$ spectrum as well as peaks with the probe tip whereas, at the tipless condition, clear double peaks are observed. The simulation result ensures that the MOLE probe exhibits a clear and simple S${}_{11}$ spectrum and as a result, easy analysis to infer electron density ($n_{e}$) just by using the equation, $f_{\mathrm{SWR}}$ = 6349$\sqrt{n_{e}}$. Furthermore, as the MOLE probe has a flat antenna, it is applicable for plasma monitoring installed on the vacuum chamber wall, which is the main purpose of the MOLE probe.

## 3. Experimental Demonstration

### 3.1. Experimental Setup

**Figure 6:**
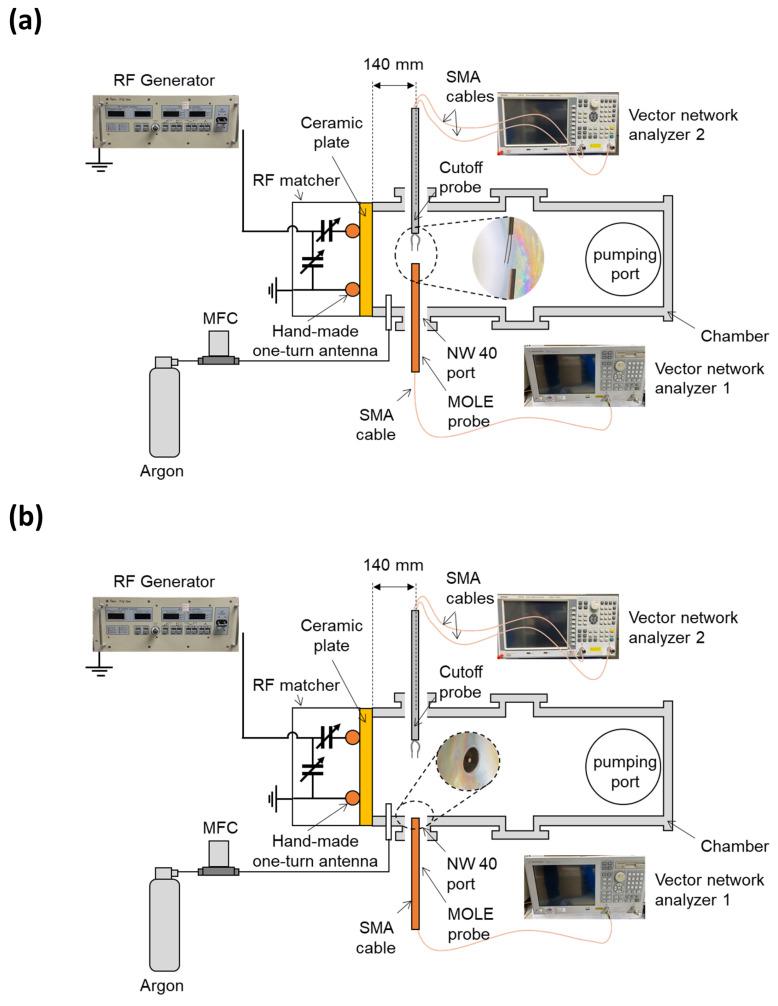
Schematic diagrams of the experimental setup for a demonstration of the MOLE probe positioned (**a**) in the center of the chamber and (**b**) on the chamber wall.

Figure 6 shows the experimental setup for a demonstration of the MOLE probe. The probe was made by cutting an RG-401 cable, a common coaxial cable. The diameter of the core conductor (copper) was 1.63 mm and the inner and outer diameters of the jacket conductor (copper) were 5.31 and 6.35 mm, respectively. The dielectric material was PTFE, of which the inner and outer diameters were 1.63 and 5.31 mm, respectively. The cutoff frequency of the RG-401 cable was 20 GHz, which is much higher than the frequency range used in this experiment, and the characteristic impedance was 50 Ω.

Inductively coupled plasma was generated by applying rf power from an rf generator (YSR-06MF, YongSin RF Inc., Hanam-si, Korea) to an in-house one-turn antenna through an rf matcher (YongSin RF Matcher, YongSin RF Inc., Gyonggi-do, Korea). A ceramic plate was used to both sustain the vacuum and propagate rf power into the gas inside the chamber. Argon gas (99.999% purity) was injected through a mass flow controller (MFC, LineTech Inc., Deajeon, Korea). A rotary pump (DS102, Agilent Inc., Santa Clara, CA, USA) was used to generate a vacuum in the chamber via pumping port, with a resultant base pressure of 0.6 mTorr.

The MOLE probe was inserted into the chamber via a NW-40 port with distance of 140 mm from the ceramic plate. One end of the probe was located in the center of the chamber (Figure 6a) and the other end was connected to a commercial SMA connector, which itself was connected to vector network analyzer 1 (E5071B 300 kHz–8.5 GHz, Agilent Inc., Santa Clara, CA, USA)) via SMA cable, as shown in Figure 6. To remove the signal loss by the SMA cable, calibration was conducted at the end of the cable with a calibration kit (85033D, Agilent Inc.).

To demonstrate the operation of the MOLE probe, a cutoff probe was inserted into the chamber via a NW-40 port opposite from the MOLE probe. The cutoff probe was connected to vector network analyzer 2 (S3601B 100 kHz–8.5 GHz, SALUKI Inc., Taipei, Taiwan) via two SMA cables. The cutoff probe can precisely measure the electron density by measuring the cutoff frequency in S${}_{21}$; details can be found elsewhere [9,46,47,48,49]. In the next section, operation of the MOLE probe at the center of the chamber is first demonstrated compared to the cutoff probe. Operation on the chamber wall is then considered, with the cutoff probe still located at the center of the chamber.

### 3.2. Experimental Results and Discussion

**Figure 7:**
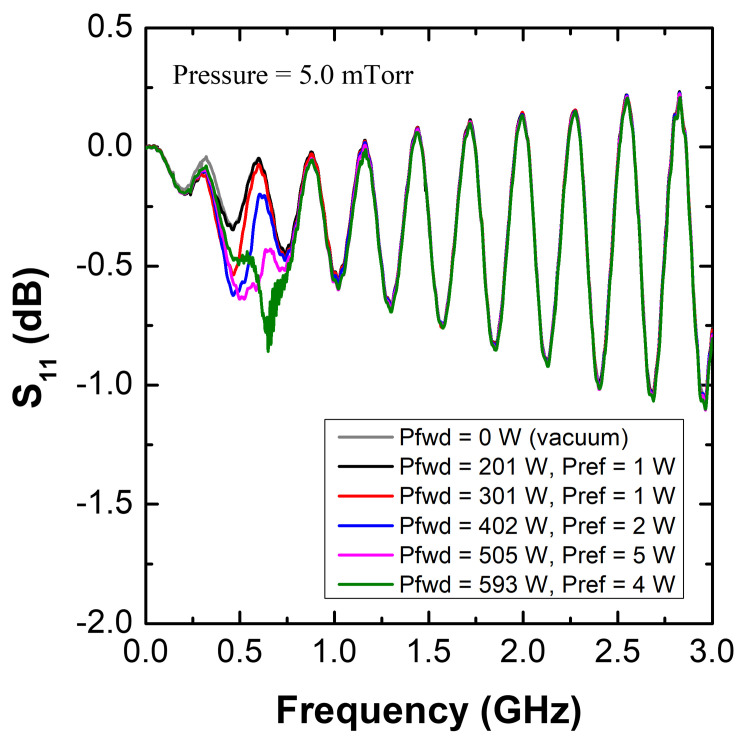
S${}_{11}$ spectra of the MOLE probe with various rf powers at a pressure of 5.0 mTorr.

**Figure 8:**
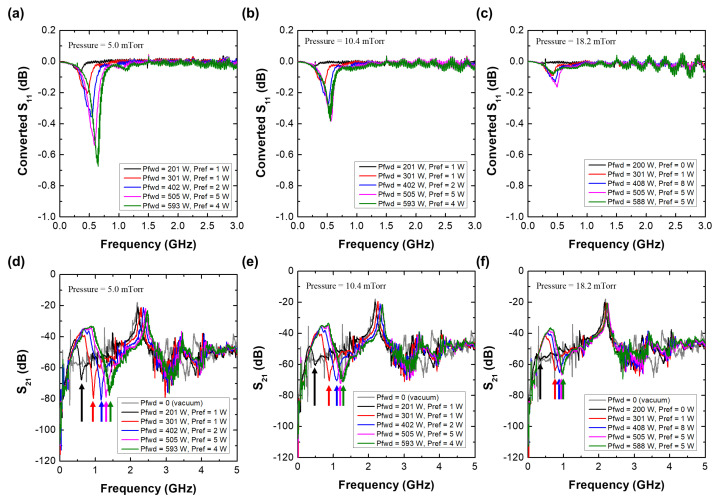
(**a**–**c**) Converted S${}_{11}$ spectra [defined as S${}_{11}$(plasma)–S${}_{11}$(vacuum)] of the MOLE probe. (**d**–**f**) S${}_{21}$ spectra of the cutoff probe at various rf powers (201–593 W) and pressures (5.0–18.2 mTorr).

Figure 7 shows the S${}_{11}$ spectra of the MOLE probe with various rf powers at 5.0 mTorr. A peak appears near 0.5 GHz with the probe at various rf powers (201–593 W) and pressures (5.0–18.2 mTorr) depending on the rf power; based on the simulation analysis, this peak is believed to be the SWR peak, with the other peaks above 1 GHz thought to originate from the standing wave resonance formed inside the coaxial cable. Since these peaks complicate the analysis, converted S${}_{11}$ spectra [defined as S${}_{11}$(plasma) − S${}_{11}$(vacuum)] are introduced in Figure 8a–c. With these, the SWR resonance can be more clearly observed. The SWR frequency is proportional to the rf power, and as the pressure increases, the Q-factor of the peak abruptly decreases. As shown in Figure 8d–g, the cutoff frequency ($f_{\mathrm{cutoff}}=8980\sqrt{n_{e}}$) in S${}_{21}$, which is marked with an arrow at each rf power condition, is proportional to the rf power [47,48,49]. This implies that the electron density is also proportional to the rf power. Combining the findings of the converted S${}_{11}$ analysis and S${}_{21}$ analysis, the SWR frequency as well as the signal reduction are proportional to the electron density. Moreover, the Q-factor of the SWR peak decreases with increasing pressure. These trends are exactly the same as those in the simulation analysis. The dependence of the SWR frequency as well as the cutoff frequency on the rf power is further analyzed by considering the wall-positioned MOLE probe results, as follows.

**Figure 9:**
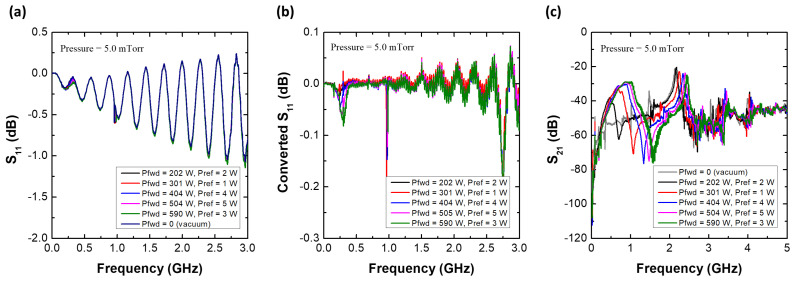
(**a**) S${}_{11}$ spectra and (**b**) converted S${}_{11}$ spectra [defined as S${}_{11}$(plasma)–S${}_{11}$(vacuum)] of the MOLE probe. (**c**) S${}_{21}$ spectra of the cutoff probe with various rf powers (202–590 W) at a pressure of 5 mTorr.

Finally, to demonstrate the operation of the MOLE probe in non-invasively measuring lateral electron density, the probe was positioned on the chamber wall as shown in Figure 6b. Here, lateral means the plasma–sheath edge near the chamber wall. Figure 9a,b show the S${}_{11}$ and converted S${}_{11}$ spectra of the wall-positioned MOLE probe at 5 mTorr. Compared to Figure 7, the SWR peak as well as the signal reduction in the S${}_{11}$ spectra are unclear since the electron density near the chamber wall is, in general, lower than that in the center of the chamber [5]. Although the SWR peak is unclear in S${}_{11}$, there is a distinct SWR peak in the converted S${}_{11}$ spectra, and this peak is proportional to the rf power. Figure 9c shows that the cutoff frequency is also proportional to the rf power. Once again, by combining these two facts, the $f_{\mathrm{SWR}}$ from the wall-positioned MOLE probe is found to depend on the electron density at the center.

**Figure 10:**
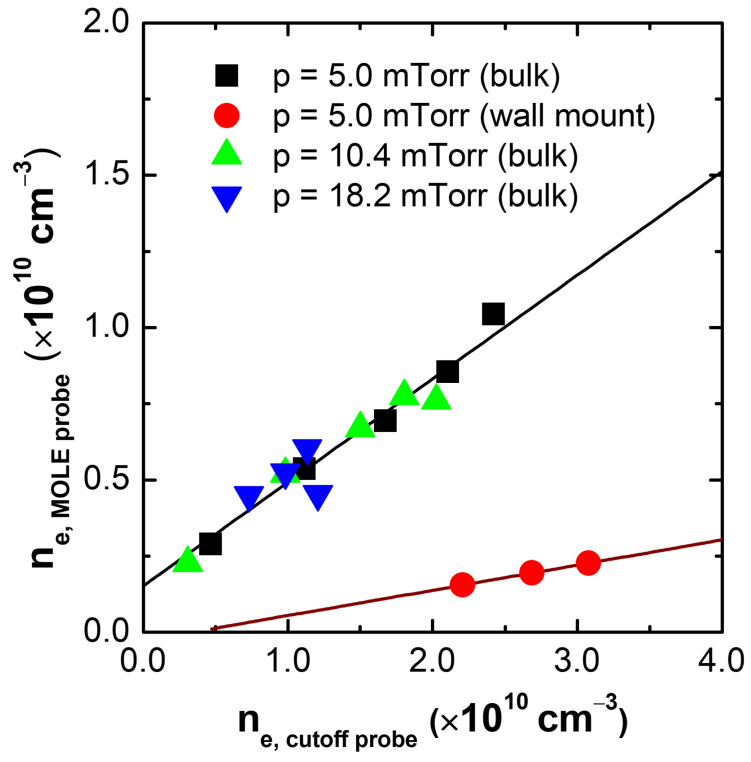
Estimated electron density from the MOLE probe ($n_{e,\mathrm{MOLE}\mathrm{probe}}$) at the bulk and on the wall over densities from the cutoff probe ($n_{e,\mathrm{cutoff}\mathrm{probe}}$) at various pressures.

Figure 10 exhibits estimated electron densities from the MOLE probe at bulk over densities from the cutoff probe at various pressures RF powers based on the measurement results of Figure 8 and Figure 9. In terms of the trend, the $n_{e,\mathrm{MOLE}\mathrm{probe}}$ measured at the bulk represents a good linearity with the $n_{e,\mathrm{cutoff}\mathrm{probe}}$, which means that the MOLE probe catches well the variation of the bulk plasma. For the wall mount MOLE probe, the $n_{e,\mathrm{MOLE}\mathrm{probe}}$ also follows the bulk variation as shown in Figure 10. Hence, through experimental demonstration with the cutoff probe, we find that the MOLE probe on the wall works well.

The MOLE probe however measures the electron density several times lower for the bulk and 10 times lower for the wall mount than the cutoff probe. This is because of thin sheath condition and the plasma non-uniformity [17,50]. Based on the simulation result (Figure 4), at a thin sheath width the $f_{\mathrm{SWR}}$ is lower than the ideal one, which means the MOLE probe estimates lower density. In fact, sheath width in the inductively coupled plasma source is known as floating sheath, which is five times the Debye length ($5\times \lambda_{\mathrm{De}}$) [51], where $\lambda_{\mathrm{De}}\left(=740\sqrt{T_{e}/n_{e}}\right)$ is the Debye length in cm and $T_{e}$ and $n_{e}$ are the electron temperature in eV and electron density in cm${}^{-3}$, respectively. Assuming the $n_{e}$ as $2\times 10^{10}$ cm${}^{-3}$ based on the cutoff probe and $T_{e}$ as a few eV [5], the estimated sheath width is the order of 0.1 mm and it surely causes the underestimation of the SWR frequency. Moreover, at low pressure, the inductively coupled plasma source has a non-uniformity as in [17] where the measured density near wall is 10 times lower than the bulk due to ambipolar potential. Hence, with considering the non-uniformity effect, the electron density by the MOLE probe on the wall is reasonable based on Figure 10.

## 4. Conclusions

The development of a microwave probe called the measurement of lateral electron density (MOLE) probe was described in this work with simulation study and experimental demonstration. Lateral as used here refers to the plasma–sheath edge near the chamber wall. Results of a 3D E/M wave simulation analysis established that the MOLE probe can measure the SWR frequency ($f_{\mathrm{SWR}}$ = 6349$\sqrt{n_{e}}$), with a theoretical measurement discrepancy up to 36.5% depending on the sheath width; especially, the discrepancy increases as sheath width decreases. Experimental demonstrations including a cutoff probe for comparison exhibits that the MOLE probe represents a good linearity with the cutoff probe at bulk as well as on the chamber wall, which means that MOLE probe can measure the lateral electron density. In conclusion, the MOLE probe positioned on the chamber wall is believed to be a useful tool to monitor lateral electron density.

## Data Availability

The data presented in this study are available on request from the corresponding author.
